# Evaluation of the antibacterial activity of the *Althaea officinalis L.* leaf extract and its wound healing potency in the rat model of excision wound creation

**Published:** 2015

**Authors:** Maryam Rezaei, Zeynab Dadgar, Ali Noori-Zadeh, Seyed Alireza Mesbah-Namin, Iraj Pakzad, Elham Davodian

**Affiliations:** 1 *Researcher, Razi Herbal Medicines Research Center, Lorestan University of Medical Sciences, Khoramabad, Iran.*; 2 *Department of Clinical Biochemistry, Faculty of Medical Sciences, Tarbiat Modares University, Tehran, Iran.*; 3 *Department of Microbiology, Faculty of Medicine, Ilam University of Medical Sciences, Ilam, Iran.*

**Keywords:** *Antibacterial*, *Wound healing*, *Althaea officinalis*, *Phytochemicals*

## Abstract

**Objectives:**

Wound is defined simply as the disruption of the biochemical, cellular, and anatomic continuity of a tissue. Plants and their extracts known as phytomedicine have immense potential for the management and treatment of wounds.

**Materials and Methods:**

Due to the undesirable side effects, in the control and treatment of the wound infections, it is recommended to use natural materials such as phytochemicals instead of chemically synthesized drugs. Thus, the aim of this research was to study the anti-microbial and wound healing potential of *Althaea officinalis L. *hydroalchoholic extract in comparison with ciprofloxacin, gentamicin, and penicillin antibiotics on clinical strains as well as pathogenic bacteria such as *Pseudomonas aeruginosa*, *Escherichia coli*, *Staphylococcus aureus*, and *Listeria monocytogenes* under in vitro conditions using micro broth dilution and disc diffusion methods. Moreover, MIC and MBC of its hydroalchoholic extract was also evaluated.

**Results:**

The results showed that although *Althaea officinalis L.* extract was not effective on gram-negative bacteria but it was efficacious on gram-positive bacteria. The extract was also tested in the form of topical administration on excision wound model in rats. In the extract-treated wounds, the wound healing percent was significantly increased in comparison with controls.

**Conclusions:**

Based on this research, herbal extract of *officinalis L.* can be a great candidate for the treatment of gram-positive infections and merits further studies.

## Introduction

Plants extracts known as phytomedicines have immense potential for the treatment and management of wounds. Phytomedicines for wound healing purposes are not only cheap and 

affordable but also are allegedly safe as hypersensitivity is rarely encountered with the usage of these extracts. Recent studies have shown that these natural agents induce regeneration and healing of the lost tissue through multiple mechanisms. However, there is a need for scientific validation and evaluation of plants of the traditional medicine before these could be recommended for healing of the wounds as drugs. Wound is defined as the disruption of the anatomic and cellular continuity of the tissue which can be generated by chemical, physical, thermal, microbial, or immunological insults to the tissue. Wound healing processes consist of integrated cellular and biochemical aspects leading to re-establishment of structural and functional integrity of the damaged tissue with regain of strength. The aim of the wound treating is to either shorten the time course required for healing or to minimize the wound undesired consequences. Attention should be paid towards discovering the agents which can accelerate wound healing processes either when they are progressing normally or suppressed by various agents such as corticosteroids, non-steroidal anti-inflammatory, or anti-neoplastics agents. Medical treatment of wound includes administration of drugs either locally (topical) or systemically in an attempt to aid wound repair. The topical agents used include antibiotics and antiseptics, desloughing agents (chemical debridement, e.g., hydrogen peroxide, eusol, and collagenase ointment), wound healing promoters (e.g., Tretinoin, aloe vera extract, honey, comfrey, benzoyl peroxide, chamomilia extract, dexpanthenol, tetrachlordecaxide solution, clostebol acetate, and the experimental cytokines). Various growth factors such as platelet derived growth factor, macrophage derived growth factor, and monocyte derived growth factor are also seem to be necessary for the initiation and promotion of wound healing. Many substances such as tissue extracts, vitamins, minerals, and a number of plant products have been reported to possess pro-healing effects. Wound healing herbals encourage blood clotting, fight infection and accelerate the healing of wounds. Current investigations is aimed to discover plant extract or new phytomedicines for treatment and management of wounds (Davidson, 1998 ▶; Lehrer, Sun et al., 1998 ▶; Robson, Mustoe et al., 1998 ▶; Stadelmann, Digenis et al., 1998 ▶). On the other hand, the importance of antibiotics cannot be underestimated in the wound healing processes because treatment of infectious diseases is almost dependent on them. Antibiotics discovery was a turning point in human history and they have revolutionized infection treatment in many respects and countless lives have been saved after their further developments. In addition to their use in the treatment of infectious diseases, antibiotics are crucial to the successful surgical operations including prosthetic and organ transplants as well. Notwithstanding all good intentions to control antibiotic usage, there is no doubt that the situation with respect to antibiotic resistance is not optimistic and antibiotic resistance creates a financial and clinical burden on health care systems. It is thought that increasing antibiotic resistance poses important risks to human health care (Levy, 1998 ▶; Quinn, 1998 ▶; Weinstein, Gaynes et al., 2005 ▶; Klevens, Edwards et al., 2008 ▶). Due to the emergence and rapid expansion of antibiotic-resistant bacteria, this topic has received considerable attention (Levy and Marshall, 2004 ▶). Unfortunately, this phenomenon is pandemic and there are no simple solutions to this problem. Bacterial pathogens, once susceptible to antimicrobial agents, are becoming increasingly resistant. In addition, new opportunistic pathogens threat hospitals and show little susceptibility to current antibiotics. Moreover, it can affect the ongoing infectious disease and aggravate the dangers associated with immune system modulators (e.g., in transplantation and anticancer chemotherapy), intubation, catheterization, and other common procedures, all of which rely on using antibiotics to overcome infections with which they are commonly associated. The common perception is that the problem was originated as exclusively associated with the use and misuse of antibiotics in humans (English and Gaur, 2010 ▶). This is true for the colonal dissemination of pathogenic bacteria with resistance mechanisms based on altering target molecules due to mutational events and strong positive selection of mutants. However, through lateral transfer of antibiotic resistance genes from other ecologically and taxonomically distant bacteria, the majority of antibiotic resistances are most likely cases of acquired resistance (Ochman, Lawrence et al., 2000 ▶).

In the current study, we evaluated the antibacterial activity of *Althaea officinalis L.* hydroethanolic leaf extract and its wound healing potency in the rat model of excision wound creation. Hydroalchoholic extract of *Althaea officinalis L.* containing phytochemicals was capable of acting as antibiotic to kill gram-positive bacteria and can accelerate the wound healing processes as well. 

## Materials and Methods

***Althaea officinalis L.*** **extract**

*Althaea officinalis L.* extraction was prepared using Soxhlet method (Redfern, Kinninmonth et al., 2014 ▶). The leaves of this plant was collected from Lorestan province, Iran and then dried in shade and subsequently grinded. The powder (100 gr) was poured into the thimbles and they were set on Soxhlet device. Distilled water was added as a solvent to 300 ml of the ethanolic mixture. Extraction was continued for 12 hours. The extract was transferred to glass containers and then in order to evaporate the remaining solvent, they were kept without the lid in the oven for 24 hours at 50 °C. For subsequent usages, the extract was kept in the 4 °C.

**Bacteria strains**

The bacteria strains which were used in this study are as follows: *Staphylococcus aureus* (ATCC 25923), *Pseudomonas aeruginosa* (ATCC 27853), *Escherichia coli* (ATCC 25922), and *Listeria monocytogenes* (ATCC7644). 

**Minimum inhibitory concentration (MIC)**

MIC test was performed in sterile 96-well plates by micro broth dilution method (5). At first, 100 µl of cultivation medium of Müeller-Hinton broth (Merck, Germany) were poured into 96-well micro plates and serial dilutions were made. In another row, in accordance with the tested bacteria sensitivity, 80 µl of ciprofloxacin, gentamicin, and penicillin antibiotics were added. In the final step of the procedure, addition of 100 µl diluted microbial suspension equivalent to a 0.5 McFarland standard was performed to all the wells. After 24 hours of incubation at 37 °C temperature, the turbidity as the evidence for the bacteria growth was recorded. According to the definition of the concentration of the last (diluted) well with no turbidity, MIC equivalent was used and extract control section, cultivation medium, and microbes were considered separately.

**Minimum bactericidal concentration (MBC)**

To test MBC, 10 µl of the three sections before MIC was separately cultivated on Müeller-Hinton agar medium. After 24 hours, the lowest concentration of the extract in which the bacteria did not grow (99% no growth) was reported as MBC concentration (5).

**Disk diffusion method**

To test the disk diffusion, the bacteria were cultivated on Müeller-Hinton agar medium. The extract discs were 6.4 mm and 20 µl of the extract was added to each disk. Afterwards, the discs were dried and the antibiotic discs were put over them. After 24 hours incubation at 37 °C, the diameters of the zones of complete inhibition were measured from the back of plates with ruler and the results of the antibiotics were compared with National Committee for Clinical Laboratory Standards (NCCLS). Ceftazidime disk and 10 µg ciprofloxacin, and 10 µg penicillin were used as control. The tests were repeated three times and the results were presented as average in the tables.

**Experimental animals**

In this study, Wistar male rats with the weight 220-250 g were obtained from animal house of neurology sciences studies of Medical Sciences of Shahid Beheshti University in Tehran. The animals were kept in a well-controlled room in terms of temperature, light, and free access to water and food. The rats were anesthetized by ketamine (50 mg per kg body weight) and xylazine (5 mg per kg body weight). 

**Excision wound creation**

The mice were anesthetized with ketamine hydrochloride solution, intraperitoneally. Hair was removed by shaving the dorsal back of the rats. Excision wounds were inflicted on the dorsal thoracic region 1–1.5 cm away from the vertebral column on either side and 5 cm away from the ear. After wound area preparation with 70% alcohol, using a sterile round seal of 2.5 cm diameter or a surgical blade or 5–8 mm biopsy punch, circular skin from the predetermined area on the depilated back of the animal was excised to its full thickness to obtain a wound area of about 280 mm^2^ diameter and 2 mm depth. Haemostasis was achieved by blotting the wound with a cotton swab soaked in normal saline. The wound was left undressed to the open environment and no local or systemic anti-microbial agents were used. The rats were distributed in three random groups as sham (received no treatment after the wound), zinc oxide ointment-treated group (as topical and in alternating days, 0.5 g based on the wound area), and *Althaea officinalis L.* hydroethanolic extract-treated group (topical as in alternating days, 0.5g, based on the wound area). Each group included 8 rats and each rat was placed in a separate cage. 

**Wound healing evaluation**

After surgery, the drug administration and the excision wound margins were traced at 2-day intervals. Measurements of the wounds were performed using digital imaging analysis for 21 days. Measurements were continued up to 21 days. Wound contraction was expressed as percentage of wound area that had healed. The wound surface was measured using template and caliper in alternating days to the full healing. 

During measurement, each rat was imaged to measure by suitable scale and image analyzer software to control the validity of manual measurement. The wound healing percentage in various groups was measured according to the following formula: 

Wound healing percentage = Wound level at the first day - Wound level on day A / Wound level on the first day × 100.

**Histological evaluation**

After wounding, the effects of Althaea officinalis L. hydroalchoholic extract on the tissue granulation, cellular density, and extension of the fibrosis of the skin was studied by immunohistochemistry using light microscope. Histological specimens of the three groups were fixed in 10% buffered formalin, paraffin imbedded, and stained with hematoxylin and eosin and the samples were studied pathologically.

**Data analysis and description**

Statistical analysis was performed using SPSS 16 software. All data are presented as means ± SEM. To compare multiple means in groups, one-way ANOVA followed by Tukey's post hoc comparison was used. Values of p<0.05 were considered statistically significant.

## Results

**MIC and MBC results**

In this study, the anti-microbial effects of hydroalchoholic extract of *Althaea officinalis *on standard strains of *Listeria*, *Staphylococcus aureus*, *Pseudomonas*, and *Escherichia coli *were evaluated. The results showed that *Althaea officinalis* extract with MIC=330±0.1 µg/ml had a bactriostatic and at concentration of MBC=660±0.2 showed a bactericide effects on *Staphylococcus aureus* but demonstrated no effects on other examined bacteria. Its MIC for *Listeria* was observed at 250±0.15 µg/ml concentration and there was no MBC (Table 1). Thus, hydroethanolic extract of *Althaea officinalis* had no effect on gram-negative bacteria.

**Table 1:** Minimum inhibitory concentration (MIC) and minimum bactericidal concentration (MBC).

**Organisms**	**MIC** **(µg/ml)**	**MBC (µg/ml)**
*Staphylococcus aureus*	330±2	2±660
*Listeria monocytogenes*	-	250±3
Escherichia coli	166±2	332±3
Pseudomonas aeruginosa	664±3	332±2

**Wound healing percentage evaluations**

**Figure 1:**
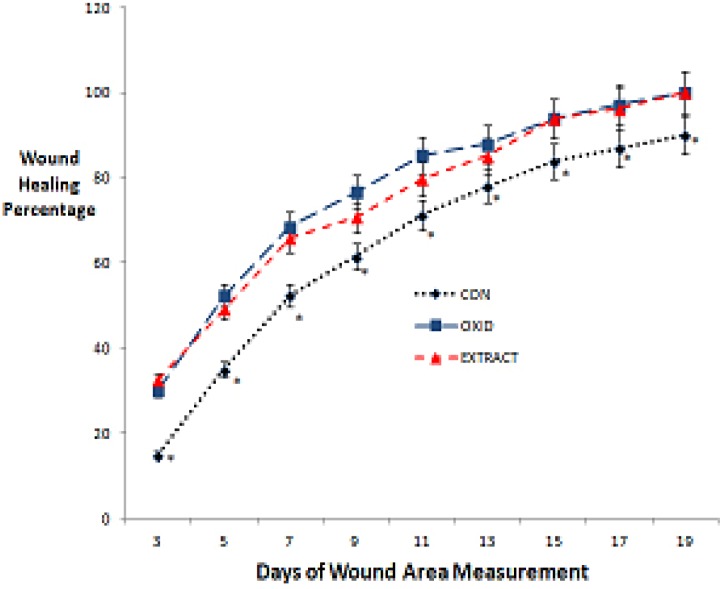
Wound healing process in the different days of wound excision repair model in the three groups. The results indicated that *Althaea officinalis L. *hydroethanolic extract-treated wounds epithelized faster in comparison with sham group as measured 6 days after treatment (p<0.05). However, the differences between the *Althaea officinalis L.* hydroalchoholic extract and zinc oxide- treated groups were not significant.

The wound healing percentages in the different days of wound excision repair model in the three groups were evaluated. The results indicated that *Althaea officinalis L.* hydroethanolic extract-treated wounds epithelized faster in comparison with sham group as measured during 6 days of treatment (p<0.05). However, the differences between the *Althaea officinalis L.* hydroalchoholic extract- and zinc oxide- treated groups were not significant statistically (Figure 1).

**Histological results**

**Figure 2:**
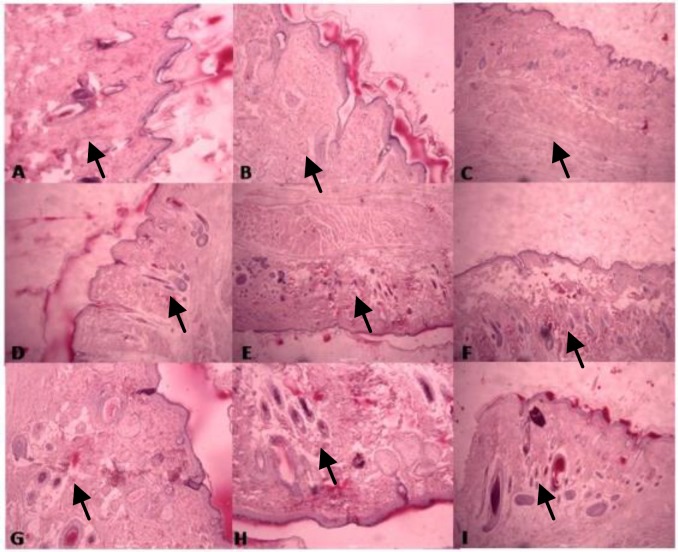
Immunocytochemistry photomicrographs of wound repair at different times of treatments in the three groups of rat model of excision wound creation A, B, and C) Wound healing after 7 days of treatment in the sham, zinc oxide, and *Althaea officinalis L. *hydroethanolic extract-treated groups, respectively. D, E, and F) Wound healing after 14 days in the sham, zinc oxide, and *Althaea officinalis L.* hydroethanolic extract-treated groups, respectively. G, H, and I) Wound healing after 21 days in the sham, zinc oxide, and Althaea officinalis L. hydroethanolic extract-treated groups.

The results showed matrix irregularity of the connective tissue and clear inflammatory reactions compared to the group receiving *Althaea officinalis* hydroalchoholic extract. Sham group had an irregular granulated tissue, more cells, and high inflammation. On 21^th^ day of the study in *Althaea officinalis* hydroalchoholic extract-treated group, the cellular density of fibrosis tissue was low, inflammation was brief, and granulation was mature in comparison with the group which received zinc oxide ointment as the scar area in zinc oxide-treated group was big and irregular but epidermal progress was consistent with *Althaea officinalis* extract-treated group. We didn't observe dermal tissue re-organization differences elements in these two groups. At the end of the treatment process, the epidermis formation was intact in both groups and the scar area was smaller as well. From the pathological aspects, the tissue samples treated with *Althaea officinalis *extract had better healing quality with regular arrangement and low inflammation density and time needed for repairing (Figure 2).

## Discussion

In the current study, the antibacterial activity of the *Althaea officinalis L.* leaf extract and its wound healing potency was evaluated in the rat model of excision wound creation. We showed that the *Althaea officinalis L.* leaf extract was effective against gram positive-bacteria and also our experiments showed the effectiveness of this extract on the wound repairing. Although, using such extracts for treating wounds is a common practice in traditional medicine, finding herbal extracts with wound healing efficacy is an increasing interest. One of the most commonly encountered clinically important impediments to wound healing is wound infections resulting from the impaired immunity and wound exposure to different microbial agents. Because the wound area is an ideal medium for the multiplication of the infecting organism, injury becomes infected. Subsequent to the development of wound sepsis, the injured tissue remains vulnerable to invasive infections of all kinds until complete epithelial repairs occur. Thus, one of the most important methods of wound care is antimicrobial therapy. Traditional medicine and current phytomedicine investigtions strongly suggest that plants have the immense potential for the management and treatment of wounds. A large number of plant extracts are used by tribal and folklore for the treatment of wounds and burns in the world. It seems that these phytomedicines induce healing and regeneration of the lost tissue through multiple mechanisms. Wound healing is the interaction of complex cascade of cellular and biochemical actions leading to the restoration of structural and functional integrity with regain of strength of injured tissues. It involves continuous cell-cell and cell-matrix interactions that allow the process to proceed in different overlapping phases and processes including inflammation, wound contraction, re-epithelialization, tissue remodeling, and formation of granulation tissue with angiogenesis (Davidson, 1998 ▶). Of the mechanisms of phytomedicines and herbal extracts and their fractions are effective inhibitions of the microbial growth, bleeding arrest from fresh wounds, and accelerated wound healing. 

The presence of various life-sustaining constituents in plants has motivated scientist to examine these plants to determine their potential wound healing properties. These active constituents promote the process of wound healing by increasing the viability of collagen fibrils and the strength of collagen fibers either by increasing the circulation or by preventing the cell damage or by promoting the DNA synthesis. On the other hand, for the eradication of infectious diseases, many attempts have been made to recognize the causes, treatment, and prevention of infectious diseases (Talei, Meshkat Alsadat et al., 2007 ▶). Based on the in vitro results, hydroethanolic extract of *Althaea officinalis L.* exerted a considerable antibacterial activity on the gram-positive strains; however, it wasn't effective against gram-negative strains. 

One of the reasons to the lack of strong antibacterial effects of hydroethanolic extract of *Althaea officinalis L.* in the present study was the low concentration of the essential oil in the extract. It is possible that this is the reason why the antibacterial effect of the extract was reduced. Another factor affecting the antibacterial effects of the extract of the plant was the extracting method and the type of solvents which were used. The extracts were obtained from the plants by different methods and solvents which may cause different antibacterial results on the specific bacteria (Nostro, Germano et al., 2000 ▶). Moreover, the enhanced wound healing potency of various herbal extracts may be attributed to free radical-scavenging action and the antimicrobial property of the different phytoconstituents present in the extract. Faster process of wound healing could be a function of either the individual or the synergistic effects of bioactive molecules. Thus, these properties give the plant extraction a specific effect on wound healing. Various studies have shown that *Althaea officinalis L.* have extensive antibacterial activity against most of the bacteria (Recio, Rios et al., 1989 ▶). 

In a similar study, the effects of this extract in the elimination of the bacteria was proved  (Gudej, 1981 ▶; Elmastas, Ozturk et al., 2004 ▶). On the other hand, wound healing processes include cell proliferation, suppression of inflammation, and contraction of the collagen tissue (Houghton, Hylands et al., 2005 ▶) and could be delayed by reactive oxygen species (Bodeker and Hughes, 1998 ▶) or microbial infection. Herbal medicine contains flavonoides which possess anti-haemorrhage properties which promotes and accelerate the healing processes of epithelial wounds by means of inhibition or activation of the enzymes that are important in the wound healing (Jahanshahi, Motahar et al., 2004 ▶). 

The results of this study showed that hydroethanolic extract of *Althaea officinalis L.* had anti-inflammatory effects and reduced the severity of the inflammation which may contribute to the wound healing as well. Plant-derived antioxidants such as phenolic acids, flavones, and flavonols, could delay or prevent the onset of degenerative diseases because of their redox properties (Govindarajan, Vijayakumar et al., 2005 ▶). This showed that hydroalchoholic extract of *Althaea officinalis L.* can be useful alone or with other antibacterial factors for treatment of some of the bacterial infections and studying its constituents is required to determine the anti-microbial specific molecule. Clinical evaluations should be used to find about the possibility of using new phytochemicals as drugs with low side-effects to treat common infections namely when there is high medicine resistance and re-infection. In conclusion, hydroethanolic extract of *Althaea officinalis L.* contains phytochemicals capable of acting as antibiotics to kill gram-positive bacteria and can accelerate the wound healing processes through other mechanisms as well. 
